# Face-to-face integrated tandem quantum-dot LEDs with high performance and multifunctionality

**DOI:** 10.1038/s41377-025-01835-9

**Published:** 2025-04-25

**Authors:** Haotao Li, Jiming Wang, Shuming Chen

**Affiliations:** Department of Electrical and Electronic Engineering, Southern University of Science and Technology, Shenzhen, 518055 China

**Keywords:** Inorganic LEDs, Quantum dots

## Abstract

The realization of solution-processed tandem quantum-dot LEDs (QLEDs) remains a technical challenge due to the limitations of inefficient interconnect layer and the damage caused by multiple solution processes. Here, we develop a high performance tandem QLED by face-to-face integrating a top-emitting QLED with a transparent QLED. The top and bottom units can be addressed independently, thereby enabling the tandem QLED to operate in series, parallel, and color-tunable modes for multifunctionality. In series mode, QLEDs demonstrate an ultra-low turn-on voltage of 3.3 V and a record-breaking external quantum efficiency of 60.7%. In parallel mode, QLEDs achieve an impressive brightness of over 4.8 × 10^6 ^cd m^-2^. In color-tunable mode, the color, brightness, and color temperature can all be adjusted with a color tuning range of 114% NSTC. Moreover, both series and parallel connections contribute to an improved stability, resulting in a T_95_ lifetime of nearly 30,000 h at 1000 cd m^-2^, which is an improvement of approximately 2.8 times over control devices. Our work offers a feasible solution for achieving multifunctional tandem QLEDs with the advantages of solution processed, high efficiency, high brightness, long lifetime and full color tunability for various light source applications.

## Abstract (web summary)

High performance and multifunctional tandem QLED is achieved by face-to-face integrating a top-emitting QLED with a transparent QLED, which exhibits a record-breaking EQE of 60.7% and can operate in series, parallel, and color-tunable modes.

## Graphical abstract

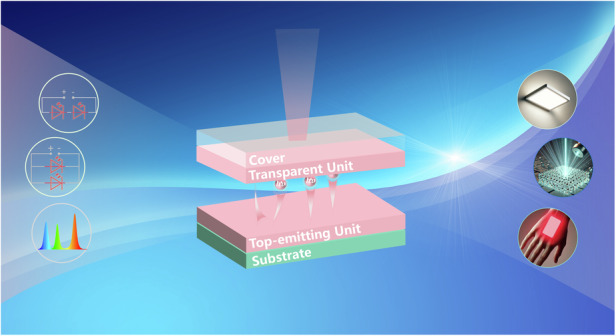

## Introduction

With the advantages of tunable emission spectra, high color saturation, and solution-processability, quantum-dot (QD) light-emitting diodes (QLEDs) are poised to become the mainstream in next-generation display and lighting technologies, offering immense market potential and significant economic benefits^1–6^. Given that the light outcoupling efficiency (OCE) of planar QLEDs is typically constrained to 20%–40%, recent advances by researchers have pushed the external quantum efficiency (EQE) of QLEDs with single light-emitting-unit (LEU) closer to the theoretical limit^7–9^. In our previous work, we achieved a red top-emitting (TE) QLED with an EQE of 37.5% without using any light extraction structures^10^. To significantly enhance the EQE of the QLED, researchers proposed the tandem structure in which two or multiple LEUs are connected by the interconnecting layer (ICL). Ideally, when a tandem structure with two LEUs connected in series, an externally injected electron can generate two photons. Consequently, the brightness, current efficiency (CE), and EQE of tandem QLEDs are twice that of a regular device, which allows for achieving high brightness at a fairly low drive current, thus offering a feasible solution for significantly prolonging the operational lifetime of QLEDs^11–13^. Considering that the EQE of the best performing QLED has surpassed 30%, the EQE of tandem QLEDs should theoretically exceed 60%. However, the maximum EQE reported for tandem QLEDs is 49%, which remains significantly lower than the expected value^13^. Furthermore, the advantage of long lifetime in tandem QLEDs has rarely been demonstrated with solid supporting data. The reasons for these discrepancies are listed below. (1) Low OCE. Tandem structures consist of multiple stacked functional layers with significant refractive index differences, leading to complex light propagation at the interfaces. Moreover, the positions of the two emitting layers are different, while the device thickness is not rigorously optimized to achieve optimal OCE for both LEUs, resulting in a low OCE that does not double compared to that of a regular device. (2) Inefficient ICL. A stable and efficient solution-processed ICL to ensure a perfect electrical connection of two LEUs has not yet been demonstrated. As a result, many tandem devices suffer from several undesirable phenomena, such as excessively high driving voltage, unbalanced carrier injection, and severe device degradation^12,14,15^. (3) Solvent damage. The increased number of layers and interfaces makes the fabrication of solution-processed tandem QLEDs a persistent challenge^11,16^. This is because the fabrication of tandem QLEDs involves multiple spin-casting and high-temperature annealing processes, which inevitably cause damage to the underlying layers, leading to performance degradation. Additionally, to avoid damaging the underlying layers, the design of tandem QLEDs must carefully consider the solvent orthogonality of different functional layers, imposing structural/material limitations on the device^15^. In 2017, our group reported the first solution-processed tandem QLED with an ICL of PEDOT: PSS/ZnMgO. Although stable white light emission was realized, the peak CE of 4.74 cd A^-1^ and the EQE of 2.0% were far lower than the theoretical values^17^. It is speculated that the ICL or other functional layers are damaged after multiple solution processing. To avoid solvent damage, the reported high-performance tandem QLEDs are predominantly based on the inverted structures, where some of the functional layers are deposited by vacuum evaporation which is incompatible with the solution process^12,18^. In addition, due to the high electron injection barrier of the ITO/ZnMgO contact and the poor conductivity of ZnMgO, tandem inverted QLEDs usually exhibit undesirable high turn-on voltage, low current density, and low power efficiency^19,20^. The above issues hinder the progress of tandem QLEDs towards practical applications, leaving a huge room for achieving an optimal tandem device. Therefore, developing a versatile, stable, solution-processed, and high-performance tandem structure is crucial for advancing QLEDs toward industrial applications.

In this study, we develop a solution-processed high-performance tandem QLED achieved by face-to-face integrating a top-emitting QLED with a transparent QLED. To avoid solvent damage caused by multiple solution processes, the top-emitting QLED and transparent QLED are independently fabricated on their own substrate, which are then assembled face-to-face and encapsulated with each other. Optically, theoretical simulations and experimental results indicate that its OCE is comparable to that of conventional tandem structure. Electrically, the top and bottom units are physically connected by their respective electrode leads, ensuring stable electrical connections and overcoming the performance degradation issues due to the inferior connection of ICL in conventional tandem devices. Benefiting from the dual substrate approach, both LEUs can be independently addressed, thereby enabling the tandem QLED to operate in series, parallel and color-tunable modes for multifunctionality. In series mode, QLEDs demonstrate an ultra-low turn-on voltage of 3.3 V and a record-breaking EQE of 60.7%. In parallel mode, QLEDs with Si/sapphire substrates can operate at a high current density of 33 A cm^-2^ and achieve an impressive brightness of over 4.8 × 10^6 ^cd m^-2^. Both series and parallel connections contribute to an improved T_95_ lifetime of nearly 30,000 h at 1000 cd m^-2^, which is an improvement of nearly 2.8 times compared to that of regular devices. High efficiency and brightness QLEDs broaden application possibilities in powerful light source for diverse applications^21–24^ including lighting, laser pumping, wearable light therapy, etc. In color-tunable mode, QLED with multiple LEUs offers a wide range of color, brightness, and color temperature tunability, showcasing a wide color tuning range of 114% NSTC.

## Results

### Comparison of OCE between conventional tandem and face-to-face integrated tandem

**Fig. 1:**
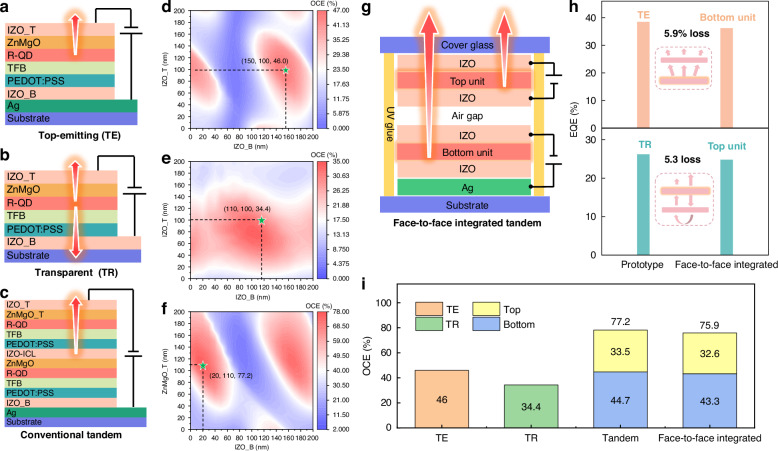
Comparison of OCE between conventional tandem and face-to-face integrated tandem. The schematic device structure of **a** TE, **b** TR, and **c** conventional tandem QLED. The OCE as a function of the distance between the emission layer and the respective electrodes for **d** TE, **e** TR, and **f** conventional tandem QLED. **g** The schematic device structure of face-to-face integrated tandem QLED. **h** The EQEs of top and bottom units in the face-to-face integrated tandem QLED, in comparison with those of TE and TR devices. **i** Comparison of the OCE in different device structures

Figure 1a–c show the schematic device structures of TE, transparent (TR), and conventional tandem QLEDs, respectively, where the latter can be considered as a vertical coupling of the former two. Optically, the QLED can be regarded as a Fabry–Pérot cavity, where the OCE is affected by the cavity length and the position of the QD layer. Transparent and conductive indium-zinc-oxide (IZO) is used as the bottom and top electrodes, which allows us to tune the cavity length without compromising the electrical performance of devices. To ensure a fair comparison, the distances between the QD and both electrodes are simultaneously varied to calculate the maximum OCE for the TE, TR, and conventional tandem QLEDs, respectively, by using a classical dipole emission model^25,26^. The thicknesses and optical parameters of the functional layers are provided in Figs. S1a, S1b, and device fabrication sections. The calculated OCEs for the TE, TR, and tandem structures are shown in Fig. 1d–f, respectively. At an optimal condition, the combined OCE of the top and bottom LEUs in the tandem structure is 77.2% (Fig. 1f), with the top LEU contributing 43.7% and the bottom LEU contributing 33.5%, respectively (detailed data for each LEU is shown in Fig. S1c). Considering that the maximum OCE of the TE structure is 46% (Fig. 1d), the average OCE (38.6%) of the two LEUs in the tandem QLED is lower than this value. To better explain this phenomenon, we calculated the power dissipation spectra (corresponding to the maximum OCE) of the TE as well as the top and bottom units in the conventional tandem device. The tandem structure includes multiple functional layers with different refractive indices, leading to a more complex light propagation at the interfaces. Consequently, the number of waveguide modes in the two LEUs of the conventional tandem is greater than that of the TE, as shown in Fig. S1d. Even worse, the bottom LEU, which is closer to the metal reflective electrode, generates a stronger surface plasmon polariton (SPP) peak induced by metal ion oscillations, further reducing the OCE of the LEU. Due to the presence of stronger waveguide and SPP modes, the LEUs in the conventional tandem structure exhibit an average OCE of 38.6%, which is 1.2 times lower than that of the TE, making the EQE of the conventional tandem far below the expected value of twice the EQE of the TE device. Furthermore, due to the stability and compatibility issues of the ICL and the inevitable multiple solution-processing steps, the efficiency and lifespan of tandem devices fall far short of their theoretical values.

To realize tandem QLEDs with truly high efficiency and long lifetime, we develop a face-to-face integrated tandem QLED based on dual-substrate assembly. As illustrated in Fig. 1g, this proposed structure consists of a TE device (bottom unit) and a TP device (top unit) that are vertically and face-to-face integrated. By extracting their electrodes, these two units can be independently controlled and can be connected in either series or parallel mode, enhancing device performance and versatility, as discussed in more detail below. Additionally, the mutual encapsulation by the substrates of both units results in extended device lifetime without the need for additional encapsulation components. The top and bottom light-emitting units are aligned by markings on shadow masks of pixel electrode to ensure high pixel overlap, as shown in Fig. S2. Previous work reported that the patterning of intermediate electrodes of vertically stacked LEUs provides the feasibility of internal electrical connections for face-to-face integrated structures, which contributes to the broadening of the potential applications of this structure^27^. With careful optical optimization and stringent cavity design, the top unit exhibits a high transmittance (~95%) while the bottom unit shows a high reflectance (~95%) without compromising the device performance, as shown in Fig. S3c, which is essential for reducing the light loss and achieving high performance tandem devices. Indeed, as shown in Fig. 1h, the bottom unit (TE device) in the tandem structure exhibits an EQE of 36.2%, which is slightly lower than 38.5% of the TE device (Fig. S3a, the histograms from different batches of 16 devices show the high repeatability of the high-efficiency QLEDs), indicating there is only a 5.9% optical loss due to the presence of the top unit. Similarly, thanks to the high reflectance (~95%, as shown in the bottom of Fig. S3c) of the bottom unit, the EQE of the top unit in the tandem ambience is only reduced by 5.3% compared to that of the TP device (Fig. S3b), as shown in Fig. 1h. Consequently, considering the EQE loss of TE and TR devices in a face-to-face integrated tandem structure, we can accurately estimate that the OCE of the bottom unit and the top unit are 43.3% and 32.6%, respectively, totaling 75.9%. As compared in Fig. 1i, the OCE of the face-to-face tandem is comparable to 77.2% of the conventional tandem, which is a prerequisite for achieving QLEDs with comparable performance. Moreover, both the bottom and the top units are fabricated on their own substrate, which not only allows them to be optimized separately, but also completely avoids damage to the underlying layers caused by solvent erosion and multiple high-temperature annealing. In addition, both units can be addressed independently, offering multiple connection modes for multifunctionality. Based on the above analysis, the face-to-face integrated tandem shows excellent optical and electrical performance, which is superior to conventional tandem and shows tremendous potential for achieving QLEDs with high performance and multifunctionality, as demonstrated below.

### Tandem QLEDs operate in series mode with record-high EQE

**Fig. 2:**
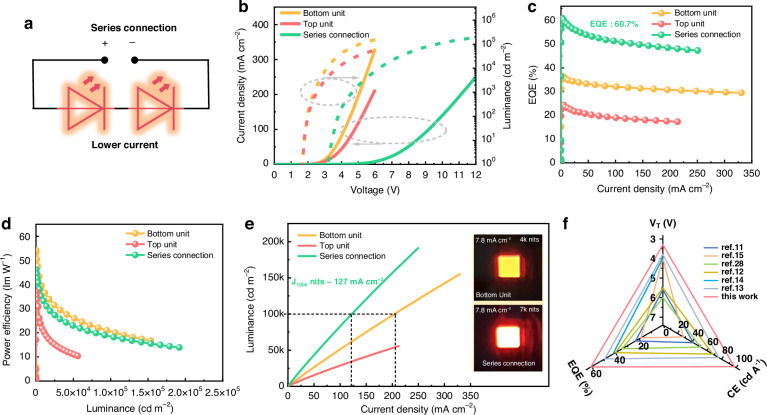
Tandem QLEDs operate in series mode. **a** The schematic diagram of electrical connection in series mode. The **b** J-V-L, **c** the EQE-J, **d** the PE-L, and **e** the L-J characteristic curves of the top and the bottom units driven in both independent and series modes. **f** Radar chart summarizing the device performance of this work and references

As shown in Fig. 2a, when the tandem QLEDs operate in series mode, the brightness can be several times higher than that of regular devices for the same current. This results in significantly improved CE, EQE, and lifetime. The spectra for the top and bottom units driven in both independent and series modes are shown in Fig. S4a. It can be observed that when the device is optimized for maximum OCE, the intrinsic emission of the QDs is largely enhanced due to the constructive interference. As a result, the peak emission wavelengths of these spectra are nearly identical. The angular emission profile of the devices shown in the inset of Fig. S3a. is used to correct the physical quantities related to normal emission intensity. Figure 2b shows the current-voltage-luminance (J-V-L) characteristics of the top and bottom units driven in both independent and series modes. In series mode, the tandem QLED exhibits a very low turn-on voltage (V_T_) of 3.3 V, which is approximately the sum of V_T_ of the top and the bottom units, indicating virtually no voltage loss in the interconnect. Notably, this is the lowest V_T_ ever reported for the tandem device. As shown in Fig. S4b, at high current, the voltage consumed by the tandem device is approximately equal to the sum of the voltages consumed by the top and the bottom units, further validating the highly conductive interconnect. As shown in Fig. 2c, the device EQE in series mode reaches an impressive value of 60.7%, which is the highest efficiency ever reported, with the bottom and top units contributing 36.2% and 24.5% of the EQE, respectively, as shown in Fig. S4c^11–15,28^. As depicted in Fig. 2d, the power efficiency (PE) of the tandem is the average of that of the two LEUs, which reaches 48.3 lm W^-1^ and is also the maximum value reported so far. The significant increase in CE is also a key feature of the series device, as shown in Fig. S4d, where the CE reaches an impressive 97.1 cd A^-1^. Figure 2e shows the J-L characteristic curves of the three devices, with the slope reflecting the magnitude of the CE. The series device consumes nearly half the current to achieve a luminance of 100,000 cd m^-2^ compared to the others, which helps to expand the lifespan of devices, as will be further discussed later. The inset vividly illustrates that under a same constant current, the series device emits a significantly higher brightness. Some representative performance characteristics of the face-to-face integrated tandem QLED are compared with those of conventional tandem reported in the literature^11–15,28^. As shown in Fig. 2f, the face-to-face integrated tandem QLED exhibits significant advantages in terms of lowest turn-on voltage (3.3 V), highest EQE (60.7%) and CE (97.1 cd A^-1^). These advantages not only signify a technological breakthrough but also demonstrate its immense potential for practical applications. In addition to conventional tandem structures, this face-to-face integrated tandem structure serves as a complementary approach to significantly improve the efficiency and stability of QLEDs.

### Tandem QLEDs operate in parallel mode with ultra-high brightness

**Fig. 3:**
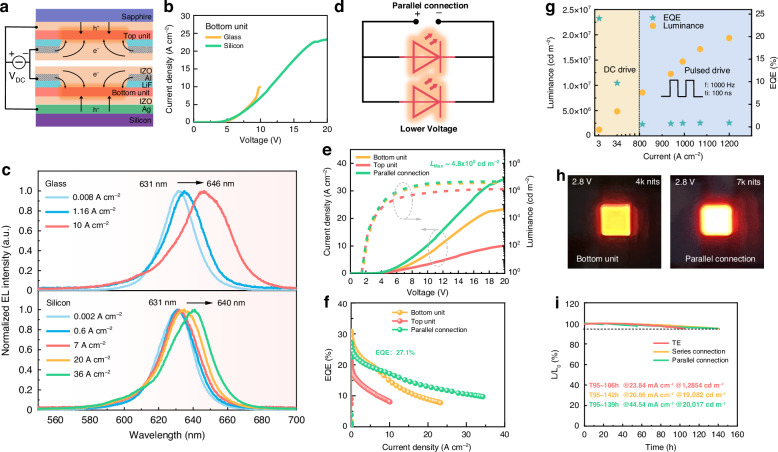
Tandem QLEDs operate in parallel mode. **a** The schematic device structure of the tandem QLED driven by parallel connection. **b** The J-V characteristic curves of bottom unit with a glass and a silicon substrate. **c** The emission spectra of bottom unit with a glass and a silicon substrate at various current densities. **d** Schematic diagram of electrical connections in parallel mode. **e** The J-V-L and **f** the EQE-J characteristic curves of the top and the bottom units driven in both independent and parallel modes. **g** L-J scatter plot of QLED under DC and pulsed driving modes. **h** Photographs of the bottom unit and the parallel device operated at the same voltage. **i** Lifetime characteristics of the TE, series, and parallel tandem devices

Low-voltage operation is crucial for achieving high brightness QLEDs^29^. In parallel connection mode, the device exhibits higher current at the same applied voltage, enabling increased brightness at lower driving voltages. To further reduce the voltage, the transparent IZO electrode is surrounded by an Al wire as a peripheral auxiliary electrode, as shown in Fig. 3a, which helps to improve the conductivity of the electrode and thus reduces the voltage dissipation in the contact leads^10^. At the high current levels required for high brightness devices, there is significant Joule heat generation within the devices, which can lead to efficiency roll-off, accelerated aging, or even catastrophic failure^30–32^. To effectively mitigate the accumulation of Joule heat within the devices, thermal management techniques should be applied^33^. This involves replacing the glass substrate of the bottom and the top units with a high thermal conductivity substrate of Si and sapphire, respectively, as shown in Fig. 3a. Due to the improved heat dissipation, device with a Si substrate can operate at a high current up to 23.3 A cm^-2^, which is significantly higher than that of the device with a glass substrate, as shown in Fig. 3b. The extent of the heat accumulation in the device can be inferred from the redshift of the emission spectra of QLEDs. This is because elevated temperatures induce lattice expansion in materials, altering atomic interactions and increasing interatomic distances, thereby narrowing the bandgap and redshifting the emission spectra^31,32^. As shown in Fig. 3c, when the current is increased from 0.0002 to 10 A cm^-2^, the emission spectra of the glass substrate device are greatly redshifted and broadened, with the peak wavelength shifting from 631 to 646 nm and the full width at half maximum (FWHM) broadening from 23 to 33 nm. Thanks to the effective heat dissipation, the peak wavelengths of the Si substrate device are only slightly redshifted from 631 to 640 nm and the broadening of the FWHM is significantly alleviated, even at an exceptionally high current density of 36 A cm^-2^. The temperature changes of the QD layer can be estimated using the Varshni equation, which describes the variation of the material’s bandgap width with temperature^32^:

1 $${{\rm{E}}}_{{\rm{g}}}\left(T\right)={{\rm{E}}}_{{\rm{g}}0}-\frac{\alpha {T}^{2}}{\left(T+\beta \right)}$$

where E_g0_ is the energy gap at 0 K; α is the temperature coefficient, and β is a parameter related to the Debye temperature of the material. The parameter variables can be referenced from ref. ^32^. The calculated temperature variations within the QD layer and the FWHM broadening for devices with different substrates are shown in Fig. S5a. With a Si substrate, the estimated temperature of the QD layer only rises to 418 K even at a high current of 36 A cm^-2^, thereby allowing the device to operate stably at high brightness.

The schematic of device’s electrical connection is shown in Fig. 3d. Figure 3e presents the J-V-L characteristic curves of the top and the bottom units driven in both independent and parallel modes. Despite the incorporation of the heat-dissipating substrates, the devices still exhibit current saturation at high voltage conditions. This is because the heat generated at high current levels can activate more interfacial and defect states, which trap carriers and then become charged centers, and as a result, these charged centers lead to increased carrier scattering and localized electric field, thereby limiting the current increase^34^. In parallel mode, the current density of the parallel device operating at the same voltage is the sum of that of the two LEUs, with a similar trend in brightness. Consequently, the parallel device achieves an ultra-high brightness of over 4.8 × 10^6 ^cd m^-2^, which is the highest reported value to date, as illustrated in Fig. S5b^5,7,33,35–37^. The ultra-high brightness QLEDs are advantageous for expanding their application scope, including in powerful light source for diverse applications^21–24^ such as lighting, laser pumping, wearable light therapy, etc. As illustrated in Fig. 3f, the EQE of the parallel device is the average of that of the two LEUs, achieving a high value of 27.1%. As mentioned before, QLEDs operating at high current usually exhibit a severe efficiency roll-off due to the increased non-radiative recombination, such as Auger recombination and thermal-induced emission quenching, as illustrated in Fig. S5c. Fortunately, the efficiency roll-off is significantly suppressed in parallel device. As shown in Fig. S4c, the EQE of the parallel device at a high current density of 20 A cm^-2^ can still maintain 50% of its initial value, which is advantageous for realizing high brightness QLEDs. To further increase the brightness, the parallel device was driven by a pulsed power supply, which allows for better heat dissipation during the intervals between pulses, thereby reducing thermal accumulation. Consequently, the device can operate at ultra-high current density and exhibit exceptionally high brightness. To measure the brightness of QLEDs under pulsed driving conditions, the signal intensity of a photodetector corresponding to a known brightness under DC driving was used as a reference. Subsequently, the pulsed signal intensities corresponding to QLEDs driven by a current pulse were detected by a photodetector and measured by an oscilloscope, and an intensity ratio relationship was established to calibrate the brightness values, as shown in Fig. S5d. Driven by pulses with a frequency of 1 kHz and a pulse width of 100 ns, as shown in Fig. 3g, the device can operate stably at a peak current density exceeding 1200 A cm^-2^ and exhibits an impressive brightness surpassing 10^7 ^cd m^-2^, corresponding to a fluence of 1.66 µJ cm^-2^ per pulse and thus enabling its use as an optical pumping source.

Figure 3h vividly illustrates the low voltage and high brightness characteristics of the parallel device. The parallel device can achieve a high brightness of 7000 cd m^-2^ at a low voltage of 2.8 V, while the series device can realize the same brightness at a low current of 7.8 mA cm^-2^. Ideally, both parallel and series connections can improve the operational lifetime of the device, as demonstrated by the lifetime test in Fig. 3i. After calibration using an acceleration factor of 1.8, the T_95_ lifetimes at an initial brightness of 1000 cd m^-^^2^ for TE, series, and parallel device are 10,509 h, 28,668 h, and 30,586 h, respectively. Compared to TE, the lifetime of series and parallel devices is improved by nearly 2.8 times. This significant enhancement in stability can be attributed to the efficient and conductive interconnection, as well as the elimination of process-related damage such as solution erosion from multiple spin-casting.

### Tandem QLEDs operate in full-color-tunable mode

**Fig. 4:**
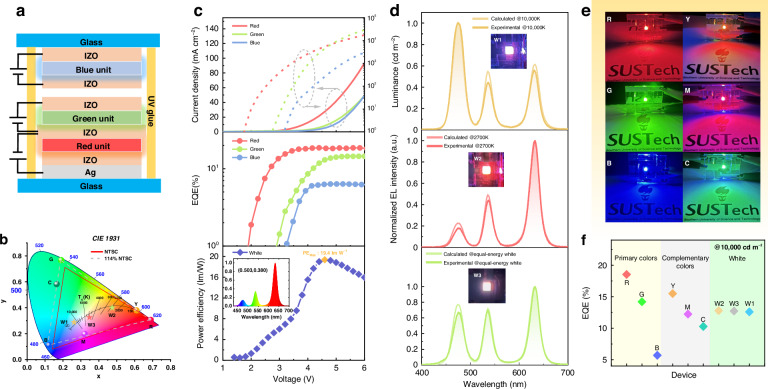
Tandem QLEDs operate in full-color-tunable mode. **a** The schematic device structure of the full-color-tunable QLED with R/G/B LEUs. **b** The color coordinates of the achievable colors by the full-color-tunable QLED. **c** The J-V-L and EQE-V characteristic curves of R/G/B LEUs under independent driving modes, together with the PE-V of R/G/B LEUs under parallel driving mode. **d** The calculated and experimental spectra of white QLEDs with different color temperatures, and the inset shows the photographs of the white lighting effects. **e** The lighting effects of the red, green, and blue primary colors, as well as the complementary colors cyan, yellow, and magenta. **f** The EQE of QLEDs with different emission colors at 10,000 cd m^-^^2^

LEDs with color-tunable functionality are in high demand due to their applications in colored and decorative lighting. To achieve a wide color tunability, three kinds of LEDs with primary colors or red, green, and blue should be placed side-by-side and controlled independently, which is quite complex. By mixing the red, green, and blue QDs in a single emitting layer, the emitting color can also be changed when the driving voltage is varied. However, such a color-tunable LED is problematic, because the color and the brightness cannot be separately controlled, and the efficiency is quite low due to the energy transfer among the closely packed red, green, and blue QDs^38–40^. Our developed face-to-face integrated tandem structure offers a smart approach to achieving high performance and color-tunable QLEDs. As schematically shown in Fig. 4a, the color-tunable QLED is obtained by face-to-face integration of a blue top unit with a bottom unit, where the bottom unit is a tandem device consisting of a red regular QLED and a green inverted QLED connected by an IZO electrode. By extracting all the electrodes, the red, green and blue QLED can be independently controlled, thereby enabling the realization of full-color-tunable QLEDs. In addition, the color, brightness, and color temperature can be adjusted separately. As shown in Fig. 4b, the color tuning range covers the color triangle defined by the color coordinates of the R/G/B emission, reaching a wide color space of 114% NTSC. A demonstration of full-color-tunable QLED is shown in the Supplementary Movie. The performances of the R/G/B LEUs under independent and parallel driving modes are illustrated in Fig. 4c. When driven in parallel by a power supply, the device reaches its peak PE of 19.4 lm W^-1^ at 4.6 V, and exhibits a reddish spectrum with a color coordinate of (0.50, 0.28), as shown in the inset. When driven independently by three power supplies, the color and the brightness can be arbitrarily tuned by changing the driving voltages of each device. As an example, we show how to realize the complementary (cyan, magenta and yellow) and white colors. To realize a specific color with a specific brightness, the brightness (and therefore the voltage according to the L-V curve) required for each LEU can be calculated, as shown in Table S1. Then, by inputting the calculated voltage into each power supply, the tandem QLED can display the required color and brightness. As shown in Fig. 4d, the obtained white spectra closely match the predicted spectra, indicating the reliability of this method. As a demonstration, we show that by varying the driving voltages, the color-tunable QLED can output white emission with different color temperatures (labeled as w1, w2, and w3, Fig. 4d), primary red, green, blue emission (labeled as R, G, and B), and complementary cyan, magenta and yellow emission (labeled as C, M, and Y), as vividly demonstrated in Fig. 4e. The color coordinates of these devices are shown in Fig. 2b. The EQE of these devices at a brightness of 10,000 cd m^-2^ is presented in Fig. 4f, with the detailed information provided in Table S1. Based on independently controllable R/G/B LEUs, the emission color, brightness, and color temperature can be adjusted individually by simply tuning the driving signal, making this device very attractive for tunable color and decorative lighting applications.

## Discussion

In conclusion, we develop a high-performance and multifunctional tandem QLED by face-to-face integrating a TE device with a TR device. The proposed tandem structure is superior to conventional tandem in terms of simple device fabrication, low solvent damage, efficient and conductive interconnect, enabling it to show a significantly enhanced efficiency and stability. Moreover, the top and the bottom units can be addressed independently, thereby enabling the tandem QLED to operate in series, parallel, and color-tunable modes for multifunctionality. In series mode, it demonstrates an ultra-low turn-on voltage of 3.3 V and a record-breaking EQE of 60.7%. In parallel mode, it can operate at an extremely high current density of 33 A cm^-2^, and achieve an impressive brightness of 4.8 × 10^6^ cd m^-2^. In color-tunable mode, the color, brightness, and color temperature can all be adjusted with a wide color tuning range of 114% NSTC. The high efficiency and brightness achieved at low current or low voltage contribute to an improved device stability, resulting in a T_95_ lifetime of nearly 30,000 h at 1000 cd m^-2^, which is an improvement of approximately 2.8 times over control devices. Our work offers a feasible solution for achieving multifunctional tandem QLEDs with the advantages of solution processed, high efficiency, high brightness, long lifetime, and full-color tunability for various light source applications.

## Materials and methods

### Materials

All materials are commercially available. CdSe-based colloidal red QDs were purchased from Suzhou Xingshuo Nanotech Co., Ltd. ZnMgO nanoparticles were purchased from Guangdong Poly Optoelectronics Co., Ltd. Poly[(9,9-dioctylfluorenyl-2,7-diyl)-co-(4,4’-(N-(pbutylphenyl))diphenylamine)] (TFB) was purchased from VOLT-AMP Optoelectronic Technology Co., Ltd. Poly(3,4-ethylenedioxythiophene)-poly(styrenesulfonate) (PEDOT:PSS) wasere purchased from Luminescence Technology Corp. IZO target materials were purchased from Hebei Gaocheng new materials Technology Co., Ltd. Chlorobenzene, and octane were purchased from Aladdin Industrial Corp. Absolute ethanol was purchased from ShangHai LingFeng Chemical Reagent Co., Ltd.

### Device structures

TE QLEDs with a structure of glass or Si/Ag (150 nm)/IZO (160 nm)/PEDOT: PSS (35 nm)/TFB (25 nm)/QDs (20 nm)/ZnMgO (70 nm)/ultra-thin Al (2 nm)/IZO (100 nm) were fabricated, and two IZOs function as phase tuning layers (PTLs) to adjust the cavity length.

TR QLEDs with a structure of glass or sapphire/ IZO (110 nm)/PEDOT:PSS (35 nm)/TFB (25 nm)/QDs (20 nm)/ZnMgO (70 nm)/ultra-thin Al (2 nm)/ IZO (100 nm) were fabricated.

The structure of bottom unit with metal wire in Fig. 3a is Si/Ag (150 nm)/IZO (160 nm)/PEDOT:PSS (35 nm)/TFB (25 nm)/QDs (20 nm)/ZnMgO (70 nm)/ultra-thin Al (2 nm)/LiF (60 nm, insulation layer)/Al (60 nm, auxiliary metal electrode)/IZO (100 nm), and the structure of top unit with metal wire in Fig. 3a is sapphire/IZO (110 nm)/PEDOT:PSS (35 nm)/TFB (25 nm)/QDs (20 nm)/ZnMgO (70 nm)/ultra-thin Al (2 nm)/LiF (60 nm, insulation layer)/Al (60 nm, auxiliary metal electrode)/IZO (100 nm).

Glass/Si/sapphire, Ag/IZO, PEDOT: PSS, TFB, QDs, ZnMgO nanoparticles (NPs), ultra-thin Al, LiF, and 60 nm Al work as substrate, electrode, hole injection layer (HIL), hole transport layer (HTL), light emission layer (EML), electron transport layer (ETL), buffer protection layer, insulation layer, and auxiliary metal electrode, respectively.

The structure of full-color-tunable QLED with R/G/B LEUs: The bottom unit in Fig. 4a is a green inverted device stacked with a red conventional device. The specific structure is glass/Ag (150 nm)/IZO (10 nm)/PEDOT:PSS (35 nm)/TFB (25 nm)/R-QDs (20 nm)/ZnMgO (70 nm)/ultra-thin Al (2 nm)/ IZO (60 nm)/ZnMgO (85 nm)/G-QDs (15 nm)/CBP (45 nm)/MoO_3_ (8 nm)/IZO (60 nm). The top unit in Fig. 4a shows a blue conventional transparent device with the specific structure of glass/IZO (70 nm)/PEDOT:PSS (35 nm)/TFB (25 nm)/B-QDs (15 nm)/ZnMgO (70 nm)/ultrathin Al (2 nm)/IZO (70 nm).

### Device fabrication

For the TE QLEDs based on glass or Si substrate, the cleaned glass or Si substrates were transferred to a magnetron sputtering system to deposit the Ag (150 nm) and IZO (160 nm) as anode at a working pressure of 0.30 Pa, a power of 50 W, an Ar flow of 20 sccm. The IZO target used here is composed of 90 wt% In_2_O_3_ and 10 wt% ZnO. Firstly, the HILs were formed by spin-casting PEDOT: PSS solution at 3000 rpm and baked at 130 °C for 20 min in the atmosphere after treating the glass/IZO and glass/ITO with O_2_ plasma for 5 min. Subsequently, the samples were transferred into a nitrogen-filled glove box to prepare the subsequent functional layers. The TFB HTLs (8 mg mL^-1^ in chlorobenzene) were spun-cast at 3000 rpm for 45 s and baked at 100 °C for 15 min. Then, the red QDs were deposited by spin-casting the QDs solution (The red CdSe/ZnS QDs were dissolved in n-octane with a concentration of 15 mg mL^−1^.) at 3000 rpm and baked at 100 °C for 5 min. Afterward, ZnMgO nanoparticles (20 mg mL^-1^ for 45 nm, 30 mg mL^-1^ for 70 nm) were spin-coated on EMLs as ETLs at 3000 rpm and baked at 100 °C for 10 min. Next, the samples were transferred to a high-vacuum evaporation chamber to deposit a buffer layer Al of 2 nm with an evaporation rate of 5 Å s^-1^ ~ 10 Å s^-1^at a base pressure of 5 × 10^−4^ Pa. Afterward, the samples were transferred to a magnetron sputtering system again to deposit the top IZO electrode (100 nm) at an environment, which is mentioned previously. In the end, the TE QLEDs were encapsulated with UV-resin and cover glass.

For the TR QLEDs based on glass or sapphire substrate, the basic process flow can be referred to above.

For face-to-face integrated structure, the effective emitting regions of the TE and TR devices overlap in vertical space, and the respective substrates are encapsulated with each other.

For the QLEDs with auxiliary metal wire electrode, prior to the deposition of the IZO top electrode, a 60 nm LiF was deposited to pattern the active area of the devices. Subsequently, a 60 nm Al was deposited on top of LiF as an auxiliary metal wire electrode. Finally, the samples were transferred to a magnetron sputtering system again to deposit the top IZO electrode, which covers both the active area and the metal wire. In the end, the QLEDs were encapsulated with UV-resin and cover glass.

For bottom units of white QLEDs, the preparation process of red LEUs referenced to TE QLEDs. After preparing the red LEUs, a solution of ZnMgO nanoparticles (50 mg/mL in ethanol) was then dynamically spin-coated on the IZO-ICL at 3000 rpm for 45 s, and annealed at 80 °C for 20 min. G-QD solution (15 mg/mL in n-octane) was dynamically spin-coated over the ZnMgO at 3000 rpm for 45 s and baked at 80 °C for 20 min. Next, the samples were transferred to a high-vacuum evaporation chamber to deposit CBP and MoO_3_ at a rate of 1 Å s^-1^ under vacuum (<5 × 10^-4^ pa). Finally, the samples were transferred to a magnetron sputtering system to deposit the IZO anode at a working pressure of 0.30 Pa, a power of 50 W, an Ar flow of 20 sccm.

### Characterizations

The thicknesses of the functional layers were measured through a Bruker DektakXT stylus profiler. The evaporation rates and the thicknesses of Al electrode were in situ monitored by a quartz crystal microbalance. The optical simulation was performed using our developed Matlab code which is based on the classical dipole emission model^25^. The J-V-L curve, EL spectrum, EQE-J curve of QLEDs were measured simultaneously by a commercialized system (XPQY-EQE, Guangzhou Xi Pu Optoelectronics Technology Co., Ltd) that was equipped with integrating sphere (GPS-4P-SL, Labsphere), fiber-optic spectrometer (XPFS1000-TEC), and a photodetector array (S7031-1006, Hamamatsu Photonics). The J-V characteristics of QLEDs were characterized by a single-channel Keithley 2400B programmable source meter under ambient conditions. The lifespan of QLEDs was measured by a ZJZCL-2 tester with the luminance calibrated with Keithley 2614B. The angular-dependent EL intensity of all the QLEDs was collected by an angle-resolved test system produced by Xi Pu Optoelectronics. The pulsed EL intensity and current were measured by an oscilloscope Tektronix TRS1102. The EQE of a pulse-driven device needs to be reasonably calculated using the following equation^8^:

$${\eta }_{EQE}=\frac{\varOmega Le}{{K}_{m}hcJ}\frac{\int I(\lambda )\lambda d\lambda }{\int I(\lambda )V(\lambda )d\lambda }$$

where *Ω*, e, *h*, and *c* are the emission solid angle, the electron charge, the Planck’s constant and the velocity of light, respectively, and *K*_*m*_ = 683 lm W^-1^ is the maximum luminous efficacy. The current density *J* is measured from the J-V characteristics using a single-channel Keithley 2400B programmable source meter under ambient conditions. *I*(*λ*) is the relative electroluminescence intensity at wavelength *λ*, measured with an Ocean Optics spectrometer (USB2000). *V*(*λ*) is the normalized photonic spectral response function. *L* is the luminance, which is calibrated by a photodetector and an oscilloscope.

## Supplementary information

Supplementary Information

Supplementary Movie Legend

Supplementary Movie 1

## Data Availability

The data that support the findings of this study are available from the corresponding author upon reasonable request.
