# Supplementary material for: Face-to-face integrated tandem quantum-dot LEDs with high performance and multifunctionality
Source: Light Sci Appl. 2025 Apr 25;14:171. doi: 10.1038/s41377-025-01835-9 (PMC12032290; doi:10.1038/s41377-025-01835-9)
Supplement: Supplementary file 1 — Supplementary Information [file 41377_2025_1835_MOESM1_ESM.docx]

**Supplementary** **Information for**

**Face-to-face integrated tandem quantum-dot LEDs with high performance and multifunctionality**

Haotao Li, Jiming Wang, Shuming Chen*

Department of Electrical and Electronic Engineering, Southern University of Science and Technology, Shenzhen 518055, P. R. China

* Corresponding author: Shuming Chen (chen.sm@sustech.edu.cn)


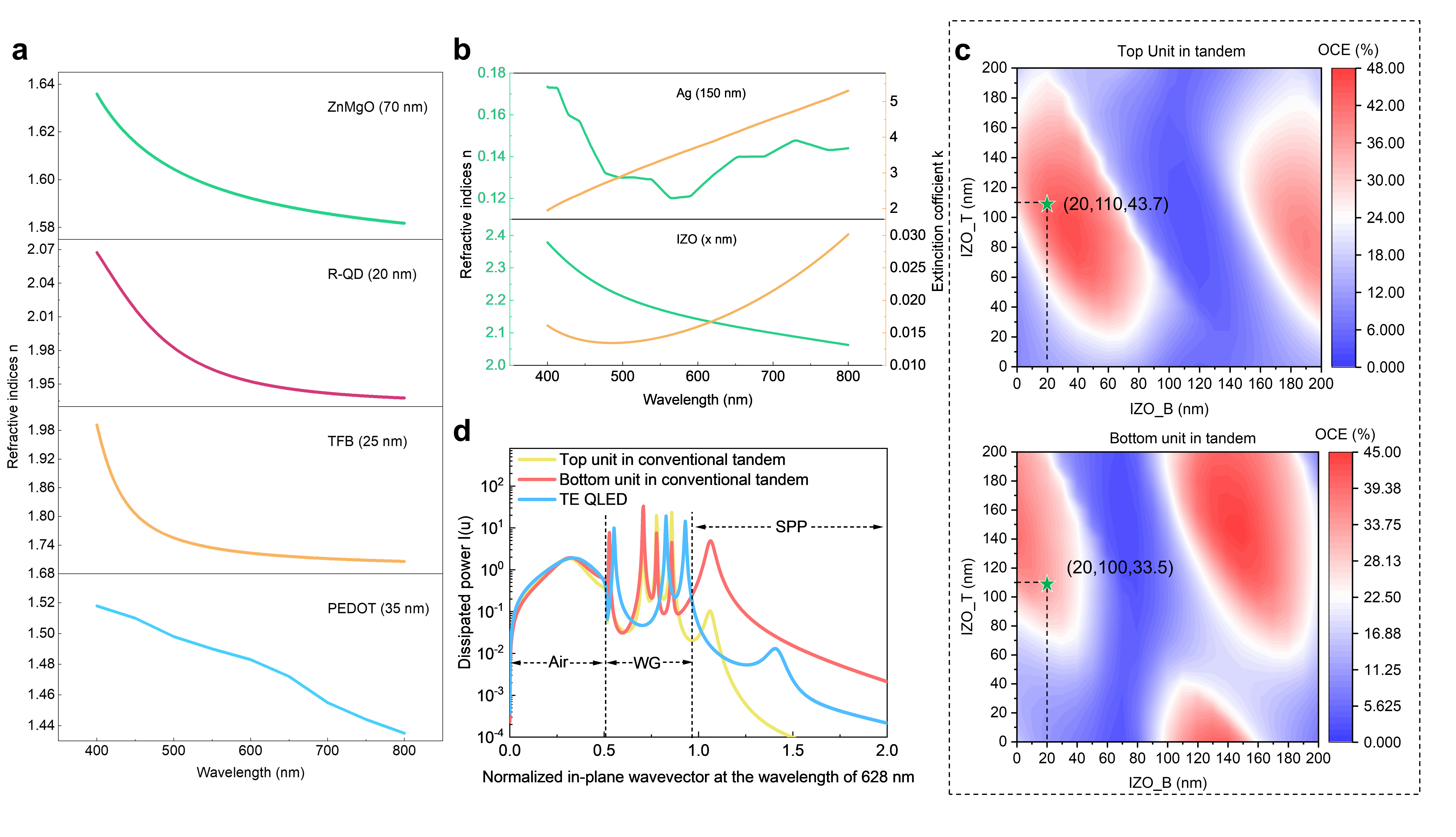


**Figure S1.** **a, b** The wavelength-dependent refractive indices of CTLs and electrodes. **c** The OCE of top and bottom units as a function of the thicknesses of both bottom and top IZOs. **d** Power dissipation spectra of TE, top, and bottom units at the wavelength of 628 nm.


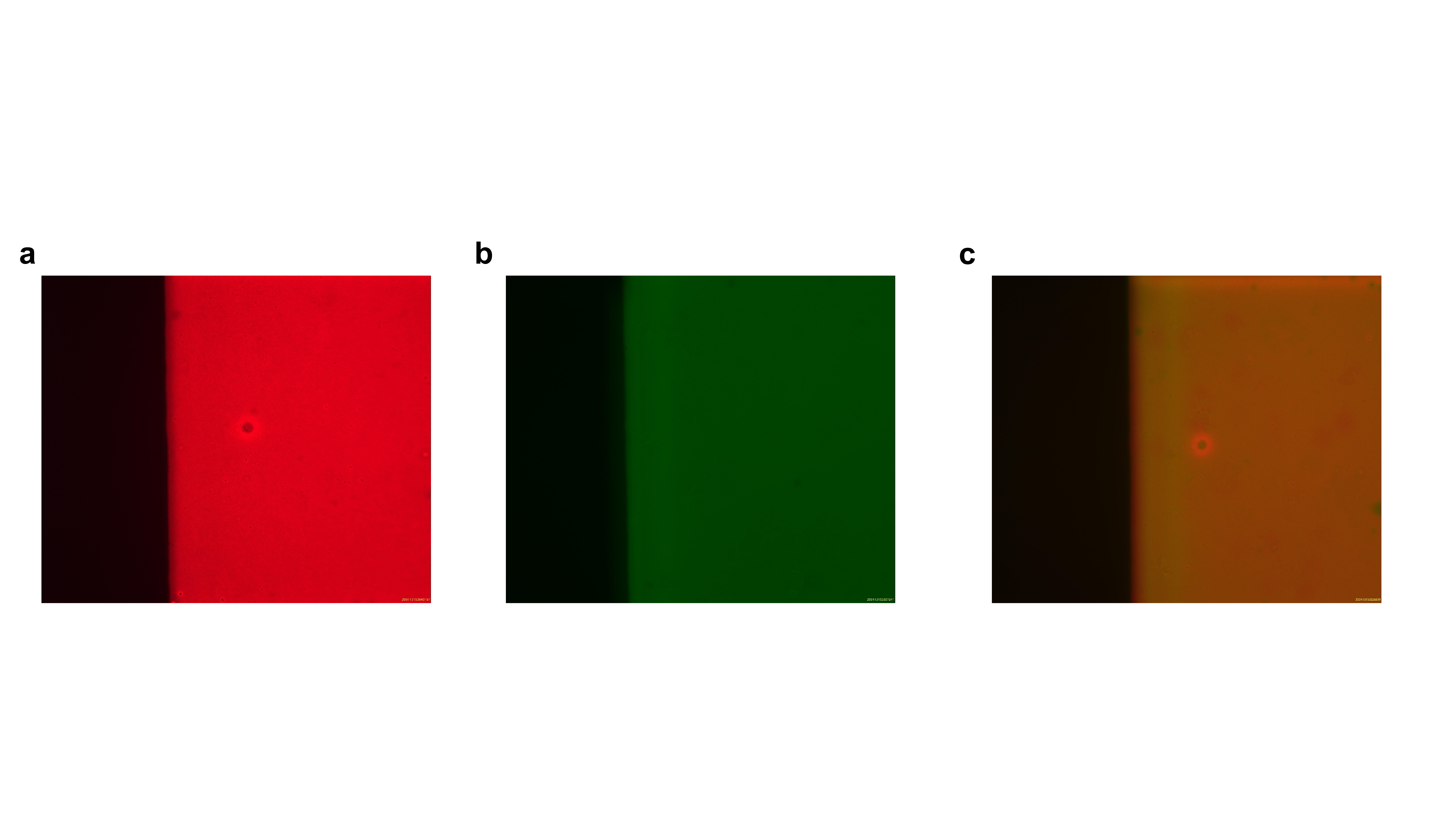


**Figure S2.** Microscope images of the pixel edges of the **a** bottom unit, **b** top unit, and **c** face-to-face integrated structure, respectively.


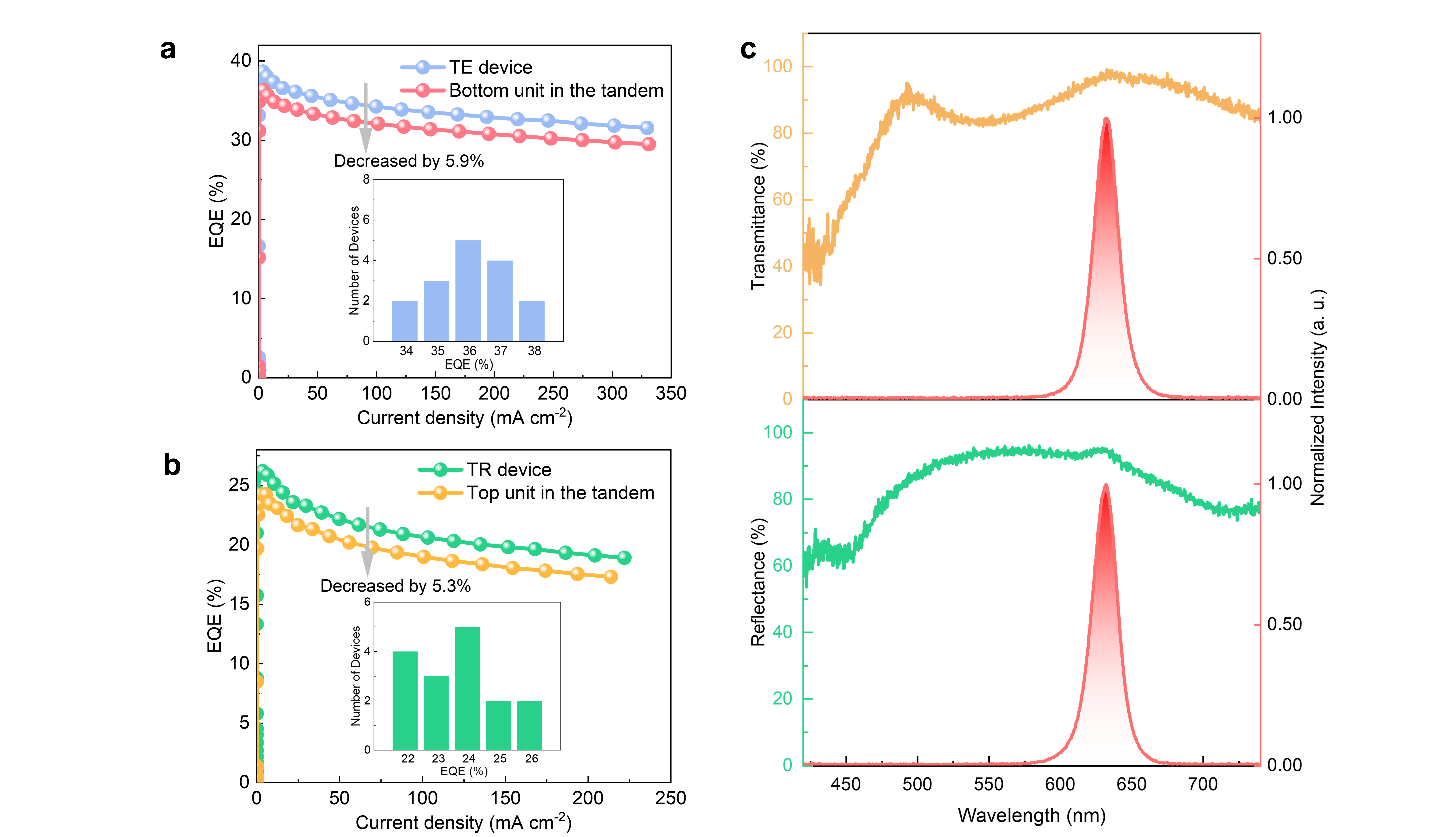


**Figure S3.** **a** The EQE-J of TE and bottom unit in the tandem. **b** The EQE-J of TR and top unit in the tandem. **c** The reflectance of TE device and transmittance of TR device.


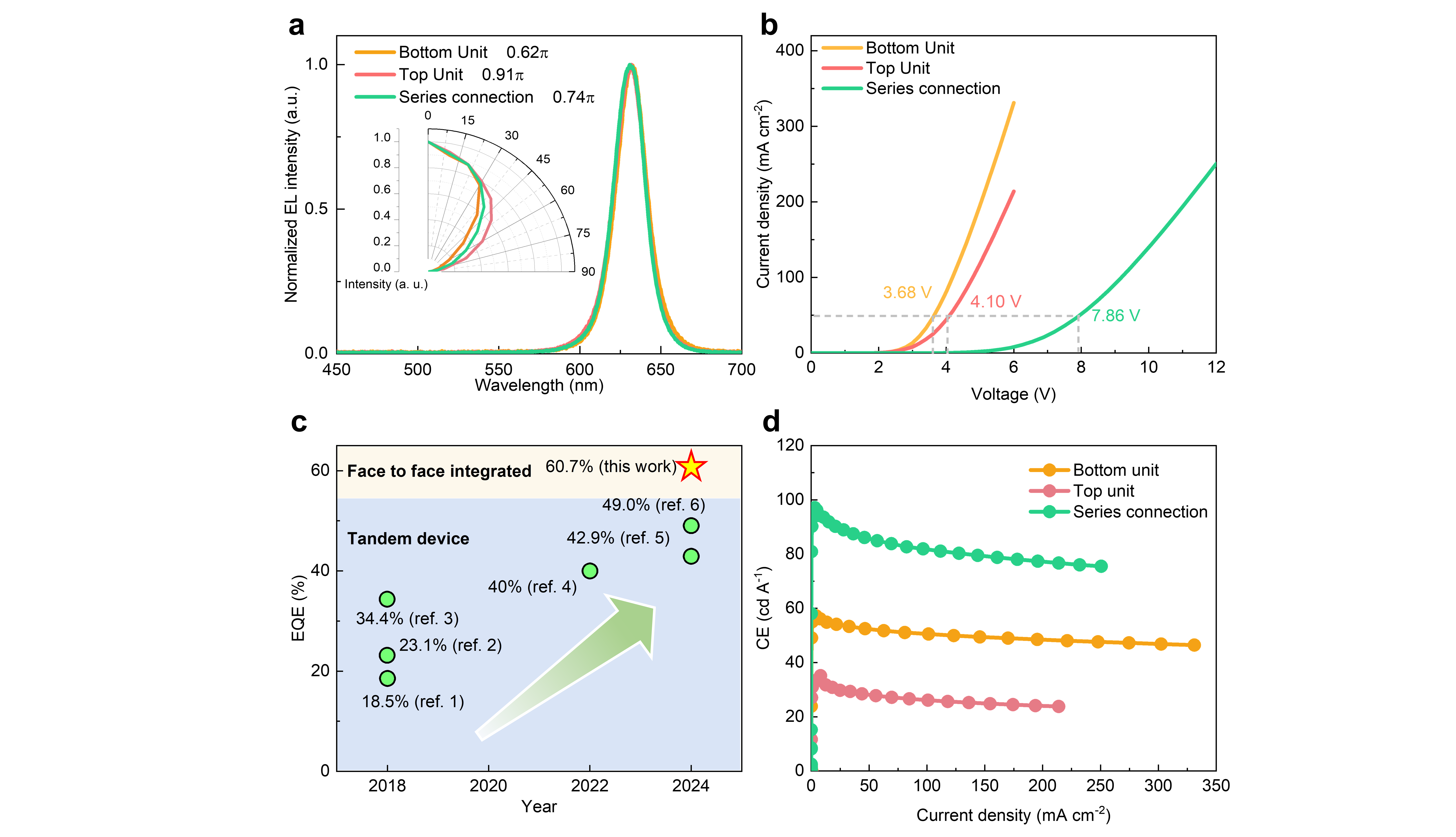


**Figure S4.** **a** The normalized EL spectra and the normalized angular dependence of EL intensity, **b** the J-V, and **d** the CE-J characteristic curves of the top and bottom units driven in both independent and series mode. **c** The development of EQE of red QLEDs over time. Data are extracted from references.


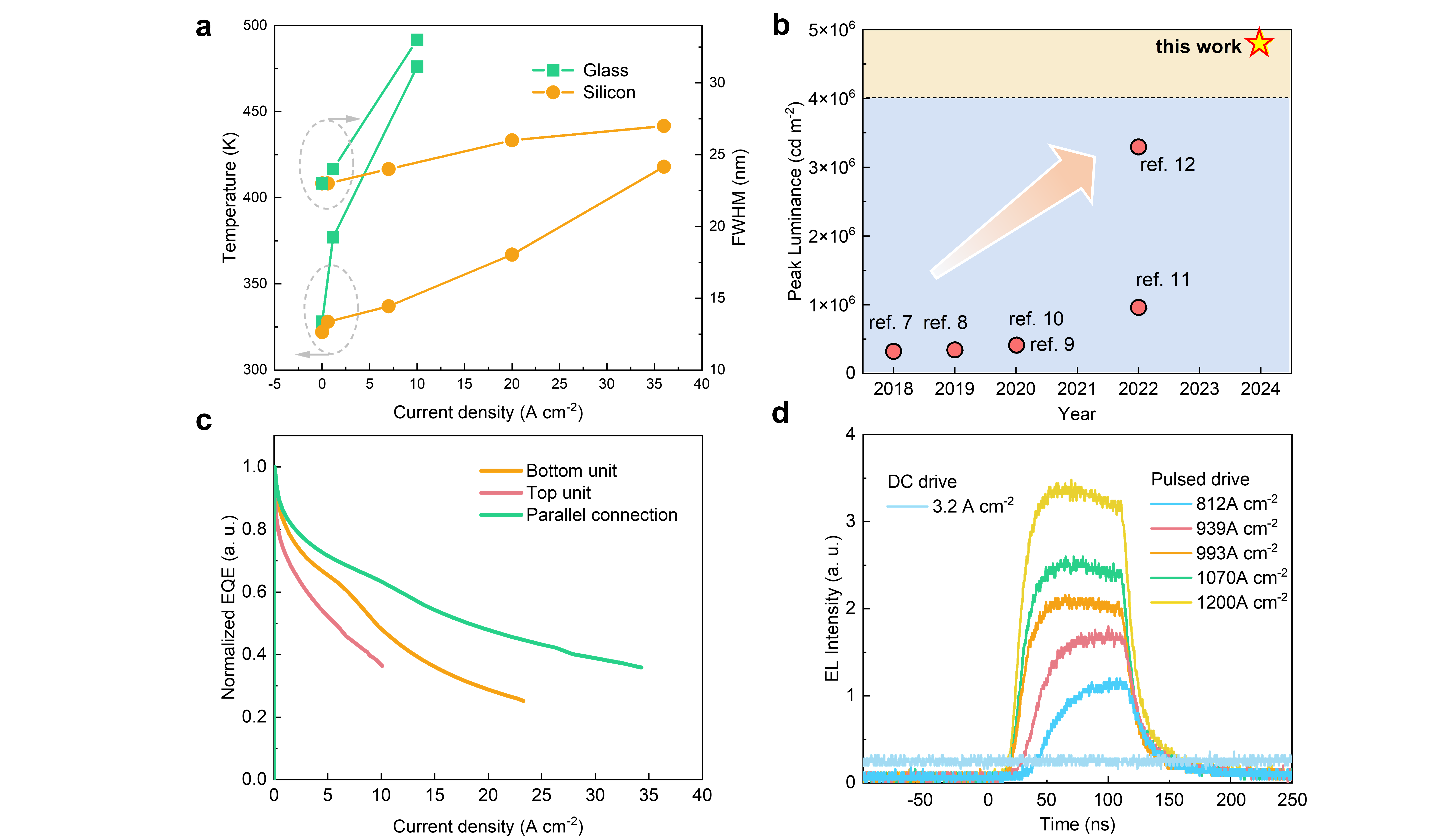


**Figure S5.** **a** The internal temperature of the QDs and the FWHM of the spectrum varying with current density. **b** The development of peak luminance of red QLEDs over time. Data are extracted from references. **c** The normalized EQE-J of the top and bottom units driven in both independent and parallel mode. **d** The luminous intensity of the device under DC and pulsed drive as detected by the photodetector.

**Table S1. Detailed performance of R/G/B LEUs for achieving different color coordinates.**

| Color | Color coordinate | U_R_  (V) | L_R_  (cd m^-2^) | EQE_R_ (%) | U_G_ (V) | L_G_  (cd m^-2^) | EQE_G_ (%) | U_B_ (V) | L_B_  (cd m^-2^) | EQE_B_  (%) |
| --- | --- | --- | --- | --- | --- | --- | --- | --- | --- | --- |
| R | (0.689,0.306) | 5.22 | 10,000 | 18.54 | / | / | / | / | / | / |
| G | (0.190,0.767) | / | / | / | 5.10 | 10,000 | 14.2 | / | / | / |
| B | (0.121,0.111) | / | / | / | / | / | / | 8.00 | 10,000 | 5.72 |
| Y | (0.610,0.379) | 4.80 | 5,591 | 18.28 | 4.60 | 4,409 | 13.01 | / | / | / |
| M | (0.321,0.197) | 4.78 | 5,456 | 18.34 | / | / | / | 6.32 | 4,544 | 6.14 |
| C | (0.207,0.582) | / | / | / | 5.10 | 9,046 | 14.2 | 5.12 | 960 | 6.34 |
| W1 | (0.254,0.239) | 3.95 | 2,340 | 18.15 | 4.72 | 5,280 | 13.4 | 5.84 | 2,380 | 6.33 |
| W2 | (0.448,0.407) | 4.38 | 4,210 | 18.63 | 4.75 | 5,230 | 13.4 | 4.88 | 560 | 6.24 |
| W3 | (0.333,0.333) | 4.12 | 3,000 | 18.48 | 4.72 | 5,490 | 13.4 | 5.50 | 1510 | 6.30 |

**References**

1. Jiang, C. B. *et al.* Fully solution-processed tandem white quantum-dot light-emitting diode with an external quantum efficiency exceeding 25%. *ACS Nano* **12**, 6040-6049 (2018).

2. Zhang, H., Chen, S. M. & Sun, X. W. Efficient red/green/blue tandem quantum-dot light-emitting diodes with external quantum efficiency exceeding 21%. *ACS Nano* **12**, 697-704 (2018).

3. Su, Q. *et al.* 73‐4: tandem red quantum-dot light-emitting diodes with external quantum efficiency over 34 %. *SID Symp. Dig. Tech. Pap.* **49**, 977-980 (2018).

4. Wu, Q. Q. *et al.* Efficient tandem quantum-dot LEDs enabled by an inorganic semiconductor-metal-dielectric interconnecting layer stack. *Adv. Mater.* **34**, 2108150 (2022).

5. Zhou, T. Y. *et al.* High-performance tandem quantum-dot light-emitting diodes based on bulk-heterojunction-like charge-generation layers. *Adv. Mater.* **36**, 2313888 (2024).

6. Yuan, C. X. *et al.* Very stable and efficient tandem quantum-dot light-emitting diodes enabled by IZO-based interconnecting layers. *Nano Lett.*, **24**, 7541-7547, (2024).

7. Lim, J. *et al.* Droop-free colloidal quantum dot light-emitting diodes. *Nano Lett.* **18**, 6645-6653 (2018).

8. Song, J. J. *et al.* Over 30% External quantum efficiency light‐emitting diodes by engineering quantum dot‐assisted energy level match for hole transport layer. *Adv. Funct. Mater.* **29**, 1808377 (2019).

9. Shen, H. B. *et al.* Visible quantum dot light-emitting diodes with simultaneous high brightness and efficiency. *Nat. Photonics* **13**, 192-197 (2019).

10. Rhee, S. *et al.* Tailoring the electronic landscape of quantum dot light-emitting diodes for high brightness and stable operation. *ACS Nano* **14**, 17496-17504 (2020).

11. Fang, Y. F. *et al.* Highly efficient red quantum dot light-emitting diodes by balancing charge injection and transport. *ACS Appl. Mater. Interfaces* **14**, 21263-21269 (2022).

12. Lee, T. *et al.* Bright and stable quantum dot light-emitting diodes. *Adv. Mater.* **34**, 2106276 (2022).
