# Supplementary material for: Face-to-face integrated tandem quantum-dot LEDs with high performance and multifunctionality
Source: Light Sci Appl. 2025 Apr 25;14:171. doi: 10.1038/s41377-025-01835-9 (PMC12032290; doi:10.1038/s41377-025-01835-9)
Supplement: Supplementary file 2 — Supplementary Movie Legend [file 41377_2025_1835_MOESM2_ESM.docx]

**Supplementary Movie**

**Supplementary Movie 1.** Demonstration of color tuning for full-color QLED.
